# Supplementary material for: Acceptance and compliance with micronutrient powder (MNP) among children aged 6–23 months in northern Nigeria
Source: PLOS Glob Public Health. 2022 Oct 17;2(10):e0000961. doi: 10.1371/journal.pgph.0000961 (PMC10022258; doi:10.1371/journal.pgph.0000961)
Supplement: S1 Dataset — (ZIP) [file pgph.0000961.s008.zip › S8_Dataset/In-depth Interviews.zip!/In-depth Interviews/040715_A_SO_BA_2_CG.pdf]

INDEPTH INTERVIEW (IDI) WITH MALLAMA MEDINATU ABUBAKAR OF GALANGWA COMMUNITY, SONG L.G.A ON 04/07/2015

Introduction

My name is Medinatu and I'm married with three children. This family is an extended one because we have relatives residing with us, though most of them are on visitation. Presently, I have one daughter currently on MNP feeding and she's 13 months of age. My husband is a farmer and we equally assist him with the farm work. We are farmers by occupation and I sale farm product harvested from our farmland at the market as well. We have to combine both so as to generate income to meet our daily needs. Food for young children is never a challenge because they feed on what the rest of the household feed on as well and in respect to my household I believe my children don't starve but get feed whenever it's required. But for children who are about to stop breastfeeding, introducing them into food could be challenging since few food available is acceptable to them. Due to this we have use milk to compliment the food we farm. What we buy is milk to support the kunu and breast milk we give to infant. I was told at the hospital that milk is rich for infants and they even prefer to accept liquid foods. The food available for mixing the MNP is the kunu which are in various varieties and I don't have challenges accessing the kunu since we cultivate maize and guinea corn. For the girl feeding on MNP, she eats our local food as well but in small quantity. It's like an introduction into the food she'll be depending on for livelihood. Since she's not taking it in large quantity, we have to compliment with the milk and kunu and atime a l have to breastfeed which is very little because we encourage a full year breastfeeding.

Tea is a liquid food normally taken in the morning and people prepare it in various forms. It is prepared by boiling water and the tea ingredient is the added to it. The ingredient comprises of milo or bournvita or lipton and sugar along with milk may be added to it depending on the taste or ability of the person taking it. I don’t give my daughter tea because I believe sugar isn’t good for her. Caregivers give the children tea mainly to serve as food since children love liquid and sweet things. The tea has become one of children favourite these days.

Experience with MNP during trial

I believe the MNP is responsible for her improved health. She now feels strong, playing in most cases and her teeth are growing out now. She has added weight as well. The MNP powder is easy to use because it doesn’t involve swallowing of tablet but to be mixed with common food that is easily accessible and easy to prepare. It mixes well with the kunu and its presence in kunu can hardly be detected. So children simply consume the MNP like their meal without detecting its presence in meal.

I’m a trader couple with farming activities, so I have tight schedules. Initially I use to leave her morning feeding in the hands of my sister in-law staying with us but to ensure the MNP is used, I have to stay around till she wakes up from sleep so that I can feed her with the kunu containing the MNP. In trying to meet up with this, my trading and farming activities have to wait a little bit. Her health is much better than anything else that is why I’m giving her much audience than other things.

I don’t have problem with storage of the MNP. It’s stored in a bedroom cupboard free from rodents or intruders. Those having problem with storage should look for a closed container or cupboard and place it in areas or angles that only the caregiver has access to it. With such

practice, both intruders and rodent won't have access to the MNP as well as moisture which can damage drugs.

The side effects observed using the MNP so far is on issue involving diarrhea. When we started giving her the MNP, she used to pass out frequent stool and it used to be water. This diarrhea was much when we started giving the MNP to her. Though it's subsiding now compared to the initial days when we started giving her the MNP. Though before I started given her the MNP, she does suffer from diarrhea once in a while but that associated with the MNP was different and it came suddenly which lasted for about three days and subsided very well. When it started, I gave her some herbal preparations to treat the diarrhea like I usually do but I didn't observe any changes so I had to stop with the advice of the nurses at the hospital and on its own it subsided or I can say it has stopped since she suffer from diarrhea less that she used to before she started taking the MNP.

I have no problem with remembering to use the MNP. I've not missed any day without giving her the MNP. My advice to people with issue of forgetfulness is to have their spouse as reminders and be determined for the good of their child. Since I started observing some positive experiences while using the MNP, it gave me courage to continue using the product. Forgetfulness majorly has to do with excessive work load by the mothers. So, I advise mothers with forgetful attitudes to seek for domestic assistances which can be in the form of relatives staying with them.

Childs feeding behavior and response to MNP

I mix the MNP only with kunu which are of various types. Sometimes I prepare the kunun gyada while other times it might be kunun masara or kunun dawa. The procedures for the

preparation of the kunu are similar. It involves making a paste of the kunu and add a boiling water and stir till it solidifies. I usually add the MNP into the prepared kunu which is ready to be served and stir it to have an even mixture before giving it to my daughter feeding on MNP. I don’t think she recognizes any differences between the kunu with MNP and that without MNP. And since I started giving, there was never a day she rejected the kunu instead she increased her rate of consumption of kunu and feeding in general. Generally in my house we normally take kunu during morning hours, so I give her the kunu along with the MNP mainly during morning hours as well. She now eats a lot, demanding for food outside her meal time and the quantity of food consumed has increased. I guess the increased feeding contributed to her recent increase in weight.

Sharing of MNP

Sharing of MNP could be in the form of siblings or household member eating the food containing the MNP together with the child MNP is meant for. It could also be in the form of giving your neighbor part of the MNP sachets to use for her child. Sharing could also be in the form of giving the remaining food containing the MNP to somebody else in the household to drink after feeding by the child MNP is meant for. I did not observed any food sharing related to the MNP because I personally feed my daughter taking the MNP and I make sure its not shared. No leftover on food containing the MNP because I measure quantity of food she can consume to avoid remains. I simply fetch a little of the kunu and mix with the MNP for her to consume. When that is done, she’ll go to join her siblings to continue her feeding because my children love to play together and they tend to consume more food when they eat together. I’ll prefer the MNP to be increased above 60 days because I want my daughter to remain healthy always because the MNP has been a great help since she does not fall ill frequently as before she started taking the product.

Perception of MNP

I perceived the MNP as a product good enough to use to improve the general health of the baby. At the hospital, they normally recommend several drugs to cure certain illnesses for children. I see MNP preventing such illnesses and due to this, it's better MNP is made available instead prevent illnesses that will warrant excessive drug consumption. Generally I see the MNP as a drug because it perform the function other drug do and its issued by health personnel and also people received advice from the hospital on issues related to the MNP.

Willingness to pay for MNP

I'm willing to pay for it because I've seen it's importance on my child. I can afford 500 naira for a packet.

I'll buy the MNP if it's to be sold because I've seen its advantages on my child and its worth using for the good health of my child. We buy drugs to treat fever, vomiting, stomach pain and others from chemist to treat our children. Since this product has reduced such instances, I will be able to pay for it depending on the amount placed on it. If you look around our community, you will know that it's a rural setting and people rarely can meet up with their family responsibilities. We are farmers and the business is not lucrative at all but we have to keep doing it to sustain ourselves and plan for the future of our children. If the drug is to be sold, I prefer it should be placed at the hospital because if left with the chemist, they will make it expensive and unaffordable to us. I can go to the drug store to purchase it for my child's use. On amount I'll be willing to buy the MNP, I prefer little charges should be placed on the drug to make it affordable. As for me, I'll be able to pay 500 Naira per packet containing the 30 sachets inside. If a drug can take me one full month for the benefit of my daughter, I don't think it's too much even though we are not that buoyant.

Social marketing of the MNP

For effective use of the product, a general section should be made at various meeting points especially by nursing mothers were information will be disseminated to them on the importance of the MNP and equally demonstrate on how to use the product because writing it on paper will not be effective to pass information since most of us are not that educated and can’t read and write. The best venues to use as the meeting points are the clinic days for nursing mothers were they bring their sick children for treatment and immunization as well. Also, the women section of various religious affiliations and various women association meetings. The doctors, traditional leaders and my husband influence my decision especially on health related issues regarding my child. My husband is the most influential because he has the final say relating to my child but we consider the doctor’s advice on health issues very important. On young child feeding, we normally have women groups were we share ideas on matters relating improving the health of our children including young child feeding and other issues as well. There we discuss on how to prepare some meals and improve our cooking skills especially on new meals using our local ingredients available to us. My husband influenced my decision regarding feeding our daughter with the MNP. Luckily, he was around when your team came to give us information regarding the product and based on the explanation he was convinced and accepted we give it a trial on our child. Even when the issue of diarrhea came resulting from the MNP, he also insisted we continue with it.

The hospital, traditional leader’s palace, peer groups or open places involving any of these groups of influential people as well as religious leaders in attendance will be preferable in disseminating health information. I trust my women association followed by the doctor’s advice or contribution on young child feeding mostly but in some cases I seek valuation

advice from people especially women I believe they are mothers. I believe that as mothers, they have the experience about bring up a child.

The radio is the best channel to reach out in mass on MNP messages. In my community, the best way to reach out is through mouth to mouth involving influential people like the village head, doctors and nurses from the hospital, the religious leaders like the imam and pastor from the place of worship in our community and various leaders of organizations. The mouth to mouth should be complimented with radio were the influential people from other communities will be interviewed. This will help build the confident or pressurize our decision makers to embrace the MNP, if they observe that other communities are accepting it as well. The flyers or posters will be an addition back up or as a reminder of any event about the MNP or what have been discussed about the product. Let's say a mother who forgets giving the MNP to her child and she sees the poster on her way going to the farm, she will suddenly remember and it may influence her decision to feed child that day.

The success of poliomyelitis program on immunization is because traditional leaders and doctors were involved to build trust on the people. They involved all the people that matters both within the community and outside. They went through challenges before gaining wide acceptance. On their case, there was a time when people heard that immunization is intended to reduce our population. Many people rejected the acceptance that period and they had to start afresh to build trust from the people. They succeeded because the government is in full support of their activity and I hope this MNP has the backing of the government.

The best channel to distribute the product is by house to house and involving the health personnel just like the cases of poliomyelitis. The team members in charge of the distribution

will move from house to house to look for those eligible to receive the MNP and will be giving what is due to them and equally use the opportunity to educate them on how to use it and the importance the MNP.

On messages to communicate, just tell the caregivers on importance of the MNP to her child and what the child is likely to benefit on using the MNP such as it will prevent fever, increase the weight of the baby and in general, it will prevent the child from falling ill always. Also, give her examples on people like me that have used the product to find out about the truth on what you've told her because people are very cautious when it comes to issues of baby.

Also, tell her the disadvantages of sharing the MNP with children or adults the product were not given. Let her know that sharing will not profit her child and the person being shared with. With all these information, i believe results will be achieved. Also the spouse should be involved to strengthen her decision and will to use the MNP for the child. my final thought is that, let the product be distributed now that people are anxious to receive it because any delay will make people to forget about many things regarding the MNP and you have to start afresh in educating people about it again. Thank you
